# Pollutants in pet dogs: a model for environmental links to breast cancer

**DOI:** 10.1186/s40064-015-0790-4

**Published:** 2015-01-22

**Authors:** Sabine Sévère, Philippe Marchand, Ingrid Guiffard, Floriane Morio, Anaïs Venisseau, Bruno Veyrand, Bruno Le Bizec, Jean-Philippe Antignac, Jérôme Abadie

**Affiliations:** LUNAM University, Nantes-Atlantic College of Veterinary Medicine and Food Sciences (Oniris), USC 1329 INRA Laboratoire d’Etude des résidus et Contaminants dans les Aliments (LABERCA), Site de la Chantrerie – CS50707, 44307 Nantes cedex 3, France; LUNAM University, Nantes-Atlantic College of Veterinary Medicine and Food Sciences (Oniris), Animaux Modèles pour la Recherche en Oncologie Comparée (AMaROC), Site de la Chantrerie – CS50707, 44307 Nantes cedex 3, France

**Keywords:** Breast cancer, Comparative oncology, Dog model, Persistent organic pollutants, Aryl hydrocarbon receptor, Etiology

## Abstract

**Purpose:**

Invasive breast carcinoma is the most common cancer in women as in non-ovariectomised pet dogs, which are already identified as a valuable spontaneous preclinical model for that disease. Geographical and time trends suggest that environmental factors may play an important role in the etiology and pathogenesis of breast cancer. Persistent organic pollutants (POPs) fit perfectly with these trends and are known to interact with hormonal receptors implicated in breast cancer subtyping. The aim of this innovating study was to evaluate the interest of the companion dog model in assessing chemical exposure and breast cancer associations, in order to identify common etiological features with the human disease in a context of comparative oncology.

**Methods:**

We monitored a hundred of molecules belonging to a large panel of POPs (dioxins, dioxin-like and non dioxin-like polychlorobisphenyls, organochlorine pesticides, brominated flame retardants, perfluorinated alkylated substances) in companion dogs diagnosed for mammary adenocarcinoma *(n = 54)* and non cancer controls *(n = 47)*.

**Results:**

All targeted chemical families were able to be detected in canine samples. We identified pollutants associated with mammary cancer belonging to the dioxin like-PCB family (notably PCB-118, -156, -105, -114) that were already pointed out in human epidemiological studies on breast cancer, and that fit with the fundamental role of the Aryl Hydrocarbon Receptor in the promotion of breast cancer.

**Conclusions:**

**S**imilarities observed in the spontaneous dog model are very helpful to progress in interpretation of human breast cancer-environment relationships. This study provides a new insight focusing on this discrete but recurrent signature.

## Introduction

Parallel observations can be made during the last decades regarding an increased environmental chemical pollution and an increased incidence of breast cancer over the same period in industrialized countries. Geographical variations, time trends, and studies of populations migrating from low to high risk areas (with migrants approaching the risk of the host country in one or two generations) clearly suggest an important role of environmental factors in the etiology of the disease. Although less commonly reported, the incidence of cancers and especially mammary (breast) cancer in pets is also increasing. Many spontaneous canine cancers are homologous to their human counterparts, regarding phenotypic but also molecular aspects (Khanna et al. 2006; Paoloni and Khanna 2008; Pinho et al. 2012). In particular for mammary cancer, biological characteristics, classifications and pathological subtypes are extremely comparable in the two species (Uva et al. 2009; Rivera and von Euler 2011; Kim et al. 2013). Breast cancer subtypes are classified depending on hormonal receptor patterns expression (Guedj et al. 2012), namely the estrogen (ER), progesterone (PR) and androgen (AR) receptors. Persistent organic pollutants (POPs) are able to interact with these hormonal nuclear receptors by penetrating cellular membranes. In a general way with some exceptions, organochlorine pesticides (OCPs), non-dioxin like polychlorobisphenyl (ndl-PCB) congeners, brominated flame retardants (BFRs) and perfluorinated alkylated substances (PFAS) are able to generate agonist or antagonist interactions with ER, AR and PR (Hamers et al. 2006; Lundholm 1988; Maras et al. 2006; Nelson 1974; Sohoni and Sumpter 1998). Dioxins (PCDD/Fs) and dioxin-like PCBs (dl-PCB) target mainly the aryl hydrocarbon receptor (AhR) (Kafafi et al. 1993; Kouri et al. 1974), which is implicated in the down-regulation of ER and AR expression at both genetic and molecular level (Ohtake et al. 2011). All these POPs are proved to be present in human as in dog blood and adipose tissues where they are stored predominantly, and to be transmitted to offspring through umbilical cord and maternal milk (Antignac et al. 2009; Den Hond et al. 2009; Fromme et al. 2010) in addition to contamination along life mainly through food chain, ingested dust or other food contacts.

OCPs were intensively produced for agriculture between 1940s and 1980s, before restrictions and bans due to their toxic and persistent characteristics in soil and food. PCBs were intensively produced between 1930s and 1980s for electrical isolation, lubrication and non-flammable capacities for various electrical and mechanic applications. Their persistence in soil and food is mainly due to contaminations from plants and inadequate disposal procedures. Their distinctive status (dl or ndl) refers to their biological mode of action on hormonal receptors, the first one mimicking that of dioxins. Dioxins are non-intentionally issued from waste incineration or some industrial processes like wood and paper, metallurgy, polyvinyl chloride (PVC) or pesticide synthesis. Despite progressive substitutions, BFRs are currently used for their flame retardant abilities in electronic devices as well as various furnishing materials including carpets and paintings. PFAS are commonly used as an anti-sticking material for cooking equipment, anti-stain treatment for textiles and surfactants in cleaning and cosmetic products.

Dogs live in the same general environment as humans, both urban and rural, whilst escaping the influence of certain carcinogens which are already formally recognized in humans, such as active smoking, alcoholic beverages or professional exposures. The limited genetic diversity seen in purebred dogs is expected to facilitate association studies on relatively small populations. Furthermore, the time-window for chemical exposure characterization should be closer to cancer induction and latency in dogs due to their shorter lifespan (about 10 years at diagnostic) and may give the opportunity of monitoring canine cohorts throughout the entire life, after however some essential issues have been solved. In this study, we monitored for the first time a large panel of POPs in companion dogs diagnosed for mammary cancers compared to control animals, in order to evaluate if the canine spontaneous model of breast cancer is able to produce results relevant to human, in a context of comparative etiology and oncology.

## 1. Methods

### 1.1. Canine population

115 female pet dogs were recruited between 2012 and 2013 in the Veterinary Hospital Center of Oniris (Nantes, France). Serum (1 ml) and subcutaneous adipose tissue (1 gram) sampling were realized during usual surgery procedures. Pet dogs have been treated humanely, and their participation during usual surgery care did not occur until informed consent of owner was obtained. Inclusion criteria were bitches aged over 4 years; exclusion criteria were neoplastic diseases other than breast, mammary dysplasia or hyperplasia (because of their unknown benign or malign outcome), and chemotherapy previous sampling. An epidemiological questionnaire was associated to each canine individual and these data were available in 90% of cases.

All these 115 dogs were used to evaluate POPs internal level of exposure in the canine population. Then, subgroups of “cancer cases” and “controls” were constituted. Cases were 54 mammary cancers (90% adenocarcinoma, 5% malignant myoepithelia, 5% sarcoma) and controls were composed of 30 benign mammary tumors (100% adenoma), whether or not combined with 17 healthy dogs (checked for absence of any neoplastic diseases).

### 1. 2. Targeted substances

115 adipose tissues samples were analyzed for dioxins, PCBs, OCPs and BFRs. Dioxins analyzed are the 17 PCDD/F congeners regulated by the European Union (EC/1259/2011). Within PCB congeners, 12 are dl-PCBs (#77; 81; 105; 114; 118; 123; 126; 156; 157; 167; 169; 189) and 6 are ndl-PCBs (#28; 52; 101; 138; 153; 180). OCPs comprise 30 compounds (or metabolites of pesticides) listed for the majority in the Stockholm convention (pentachlorobenzene, HCB, α-HCH, β-HCH, γ-HCH (=lindane), δ-HCH, heptachlor, heptachlor epoxide cis (=exo B), heptachlor epoxide trans (=endo A), γ-chlordane (=trans), α-chlordane (=cis), trans-nonachlor, cis-nonachlor, oxychlordane, aldrin, dieldrin, endrin, endrin aldehyde, endrin ketone, α-endosulfan, β-endosulfan, endosulfan sulfate, mirex (=perchlordecone), o,p’-DDE, p,p’-DDE, o,p’-DDD, p,p’-DDD, o,p’-DDT, p,p’-DDT, methoxychlor). BFRs include α, β, γ − HBCD isomers and 8 PBDE congeners (#-28, − 47, − 99, − 100, − 153, − 154, − 183 and – 209).

62 canine serum samples were also analyzed for PFAS, among which 5 perfluoroalkyl sulfonates: perfluorooctane sulfonate (PFOS), perfluorobutane sulfonate (PFBS), perfluorohexane sulfonate (PFHxS), perfluoroheptane sulfonate (PFHpS) and perfluoro-1-decanesulfonate (PFDS), and 11 perfluorocarboxylic acids: perfluorooctanoic acid (PFOA), perfluorobutanoic acid (PFBA), perfluoropentanoic acid (PFPA) perfluorohexanoic acid (PFHxA), perfluoroheptanoic acid (PFHpA), perfluorononanoic acid (PFNA), perfluorodecanoic acid (PFDA), perfluoroundecanoic acid (PFUnA), perfluorododecanoic acid (PFDoA), perfluorotridecanoic acid (PFTrDA) and perfluorotetradecanoic acid (PFTeDA).

### 1.3 Chemical analysis

#### 1.3.1. Sample preparation

Samples were transferred into individual cells in order to extract fat content by Accelerated Solvent Extraction (ASE) using a Dionex ASE 300 device. Pressure and temperature were set to 100 bars and 120°C respectively. The sample lipid weight (l.w. in % of wet weight) was calculated after using a rotary evaporation (40°C). After ASE, samples were divided into aliquots for targeting specific chemical substances. ^13^C-labelled analogues were added for quantification by the isotope dilution method. For PCBs, PCDD/Fs and brominated flame retardants, purification and fractionation are performed in three stages involving silica, Florisil and charcoal–celite columns. For organochlorine pesticides, a gel permeation chromatography was preferred to the three stages columns. For perfluoroalkyl acids, two consecutive solid-phase extraction (SPE) columns were used. A recovery standard was added to each vial for each class of compounds.

#### 1.3.2. Instrumentation

PCDD/F, PBDE, PCB and OC pesticides measurements were performed by gas chromatography coupled to high resolution mass spectrometry (GC-HRMS) using a 7890A gas chromatograph (Agilent) coupled to a JMS 700D magnetic and electric sector high resolution mass spectrometer (Jeol, Tokyo, Japan). Ionization was achieved in the electron ionization mode with 42 electron energy (eV), except for decaBDE and OC pesticides (70ev). The spectrometric resolution was set at 10,000 (10% valley), and the signal acquisition was performed in the Single Ion Monitoring (SIM) mode focusing on the two most abundant signals from each target molecular ion, or two fragment ions for OC pesticides.

HBCD and PFAS analyses were performed by liquid chromatography coupled to tandem mass spectrometry (LC-MS/MS) using an HPLC pump with a binary gradient system (Agilent Technologies, Santa Clara, CA, USA) coupled to a 6410 triple quadrupole instrument (Agilent Technologies, Santa Clara, CA, USA). Two diagnostic signals (qualifier and quantifier MRM transition respectively) were monitored for each target compound.

#### 1.3.3. Limits of detection

Limits of detection (LOD) were calculated for each congener and each sample, and corresponded to the concentration characterized by a Signal/Noise ratio of 3. Procedural blank and quality control samples were included in each series of ten to twenty samples: the signal of each compound in the blanks was checked to avoid contamination throughout the analytical procedure, and concentrations of analytes in quality control were monitored to ensure repeatability and/or accuracy.

### 1.4. Statistical analysis

Results were expressed either as a sum of congeners for each chemical family, or by congener, in nanogram (ng) or picogram (pg) per gram of lipid weight (l.w.) extracted from adipose tissue or serum samples. For PCDD/F and dl-PCB compounds, Toxic Equivalents (TEQ) were also calculated according to the World Health Organization Toxic Equivalency Factors (WHO_2005_ TEFs). Considering the independence of canine subgroups, the number of dogs in a block (n ≥ 30) and the non-normal distribution of values, Mann-Whitney was used as a non-parametric statistical test to compare characteristics and results by pair in a case/control study. Differences with p-values <0.05 were considered as significant.

### 1.5 Ethical standards

115 client-owned mammary tumor-bearing or healthy female dogs were enrolled in this study. The owners’ written consent and approval from the Oniris College of Veterinary Medicine local Animal Welfare Committee were obtained prior to inclusion.

## 2. Results

### 2.1. Epidemiological characteristics of the canine population

For the entire group of 115 female dogs, breeds were distributed in: 15% Retrievers, 15% Yorkshire, 9% others Terriers (West Highland White, Dandie, Fox, Wheaten terriers), 8% Spaniels, 6% German Shepherds, 5% Beauceron, Poodle and Dachshunds, 4% Beagle, Boxer and Lhassa Apso, 3% Doberman pinsher and Shar Peï, 14% crossed breed. Age ranged from 4 to 17 years old (mean = 11 +/- 3). Mean weight was 17.6 kg with a standard deviation of 11.2, a minimum of 1.5 and a maximum of 41.2. Canine body mass index (BMI) was estimated on scale from 1 (morbid thin) to 9 (morbid obesity), and the average score for the population was 5 (normal). Dog food was mainly dry food originated from various supermarket brands (including food industry by-products and fishmeal), supplemented by part of human food for 70% of dogs. Regarding the places where the dogs live, about 54% of dogs were coming from countryside, 30% from urban areas and 16% from downtown. Regarding physical activity, 38% of dogs were declared to have no or scarce activity (0-1h of walk per week), 22% had moderate outdoor activity (1-2h), 23% had regular outdoor activity (2-5h) and 15% had intensive outdoor activity (>5h, including hunting).

Characteristics of each subgroup used in the case/ control study (Table 1) were very similar to those of the entire group and do not constitute confounding factors, except for overrepresentation of preventive ovariectomy in healthy dogs, compared to cancer or to controls bearing benign mammary tumors. For this reason in order to avoid a potential bias, subsequent statistical analyses were performed using both control groups in parallel. A deficit for physical activity was also observed in the cancer group, but no significant difference was noticed nor for BMI, neither for the lipid content in samples (Table 1). Physical activity is further checked.

**Table 1:** **Epidemiological characteristics of « Mammary cancer » versus « Non cancer » or « Benign mammary tumor » dog subgroups (Difference with p-value >0.05 is non-significant)**

**Characteristics**	**54 Cancer** ^**(a)**^	**47 Non cancer** ^**(b)**^		**30 Benign tumor** ^**(c)**^	
**Characteristics**	**Mean ± SD**	**Mean ± SD**	**p-value** ^**(d)**^	**Mean ± SD**	**p-value** ^**(e)**^
Age (year)	10 ± 2.7	10 ± 2.5	0.104	10 ± 2.5	0.185
Weight (kg)	16.9 ± 14.4	18.5 ± 11.6	0.282	15.1 ± 10.4	0.275
Canine equivalent for BMI	5.5 ± 1.4	5.4 ± 1.1	0.714	5.5 ± 1.3	0.780
Lipids % in sample	57.6 ± 15.3	58.8 ± 15.9	0.613	58.6 ± 15.3	0.835
Lipids weight in sample (g)	0.711 ± 0.350	0.698 ± 0.353	0.826	0.741 ± 0.384	0.827
Other characteristics	Frequency	Frequency		Frequency	
Physical activity (per week)					
▪ 0-1h	47%	27%		30%	
▪ 1-2h	23%	21%		21%	
▪ 2-5h	18%	30%		30%	
▪ >5h	12%	22%		19%	
Pet food					
▪ Supermarket	83%	85%		84%	
▪ Veterinary	17%	15%		16%	
▪ Dry food	72%	73%		73%	
▪ Humid food	28%	27%		27%	
▪ + Leftovers	71%	70%		70%	
Living place					
▪ Countryside	56%	52%		55%	
▪ Urban area	23%	33%		31%	
▪ Downtown	21%	15%		14%	
Smoking owner					
▪ No smoker	68%	73%		72%	
▪ Outside	20%	18%		21%	
▪ Inside	12%	9%		7%	
Number of litter					
▪ 0	72%	64%		61%	
▪ 1	13%	17%		21%	
▪ ≥2	15%	19%		18%	
Ovariectomy	23%	47%		26%	

^a^Mammary cancer.

^b^Non cancer.

^c^Benign mammary tumors.

^d^Mammary cancer *vs*. Non cancer.

^e^Mammary cancer *vs*. Benign mammary tumors.

### 2.2. POPs internal levels in the entire group

All the targeted chemical families were detected in the studied dogs (Table 2). The 17 targeted PCDD/F congeners reach an approximate cumulative level ranging from 4 to 278 pg/g l.w. (median = 16 pg/g l.w.). The cumulated concentration levels of the 12 dl-PCB congeners ranged from 201 to 15777 pg/g l.w. (median = 830 pg/g l.w.). Considered together, the 17 PCDD/F and 12 dl-PCB congeners lead to a toxic equivalent value (WHO 2005 TEQ) of 0.5 pg/g l.w. (min. 0.1 – max 4.1), which represents a conventional way to evaluate the cumulated level of toxicity for these particular two chemical families. In terms of frequency, the less abundant congeners that are supposed to be the most toxic congeners among these specific substances were detected in at least 86% of dogs.

**Table 2:** **Quantitative determinants of internal exposure and relative contribution of main congeners for dioxins, dl-PCBs, ndl-PCBs, OCPs, BFRs and PFAS in dogs**

**Chemical family**	**n**	**Minimum**	**Maximum**	**25-75%**	**Median**	**Main congeners**
Dioxins	115	3.57 pg/g	278.29 pg/g	10-27 pg/g	15.59 pg/g	OCDD 63%
						1234678-HpCDD 24%
						123678-HxCDD 5%
						2378-TCDD 0.5%
						13 others 7.5%
dl-PCBs	115	201.07 pg/g	15776.67 pg/g	515-1545 pg/g	829.90 pg/g	PCB-118 36%
						PCB-156 26%
						PCB-189 22%
						PCB-105 7%
						PCB-157 5%
						PCB-167 2%
						PCB-114 1.5%
						5 others 0.5%
ndl-PCBs	115	0.78 ng/g	99.52 ng/g	2-9 ng/g	4.28 ng/g	PCB-180 61%
						PCB-153 31%
						PCB-138 6%
						3 others 2%
OCPs	94	0.50 ng/g	63.92 ng/g	6-15 ng/g	5.30 ng/g	HCHs 67%
						Oxychlordane 13%
						Dieldrin 10%
						HCB 5%
						Pentachlorobenzene 3%
						Cis-heptachlor epox. 2%
BFRs	115	1.63 ng/g	272.95 ng/g	9-24 ng/g	16.93 ng/g	PBDE-209 85%
						HBCD-α 9%
						PBDE-153 3%
						9 others 3%
PFAs	62	0.50 ng/ml	30.81 ng/ml	3-8 ng/ml	4.91 ng/ml	PFOS 49%
						PFHxS 25%
						PFNA 12%
						PFOA 10%
						12 others 4%

The cumulated concentration levels of the 6 ndl-PCB congeners ranged from 0.8 to 99.5 ng/g l.w. (median = 4.3 ng/g l.w.). The 30 targeted OCPs reach a median cumulated level of 5.3 ng/g l.w., with a minimum of 0.5 and a maximum of 63.9. The most abundant pesticides found in dogs are hexachlorocyclohexane (HCH) isomers (67% of total), followed by oxychlordane (13%) and dieldrin (10%). Other significant compounds are hexachlorobenzene (HCB), pentachlorobenzene and cis-heptachlor epoxide. It can be noticed that no trace of DDT isomers or related metabolites were detected in this canine population. The concentration levels of BFRs vary from 1.6 to 273 ng/g l.w., with a median value of 16.9 and a partition in 90% of PBDEs and 10% of HBCDs, approximately. Highly brominated PBDE-209 represents around 85% of the total BFR contamination, whereas lower brominated PBDE congeners (#28, 47, 99, 100, 153, 154 and 183) accounted for only 5%. The concentration levels of PFAS in serum samples varies from 0.5 to 30.8 ng/ml l.w., with a median value of 4.9 and a general partition in 74% of perfluoroalkyl sulfonates and 26% of perfluorocarboxylic acids.

**Figure 1:**
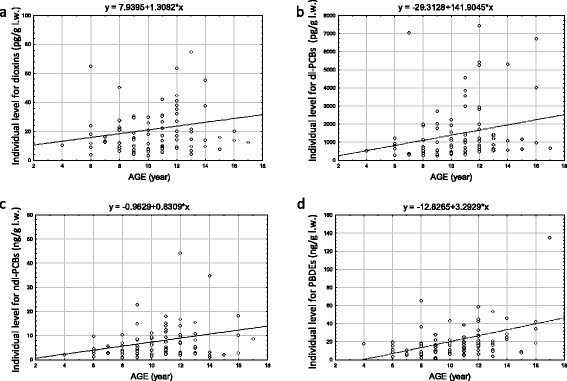
**Trends of bioaccumulation with age in dogs for (a) dioxins, (b) dl-PCBs, (c) ndl-PCBs, (d) PBDEs.**

Bioaccumulation properties linked to the age are also demonstrated (Figure 1).

### 2.3. Significant features in case-control study of canine mammary cancer

**Figure 2:**
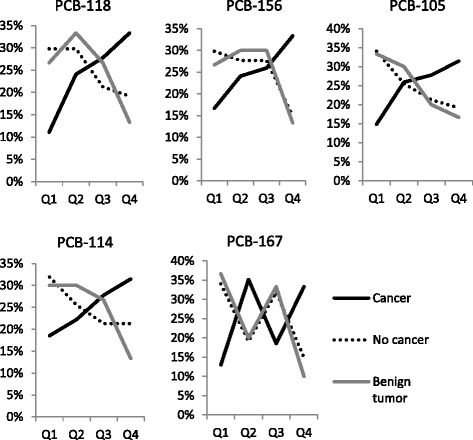
**Distribution of cancer and control cases depending on the concentration of dl-PCB congeners, grouped into quartiles.**

**Figure 3:**
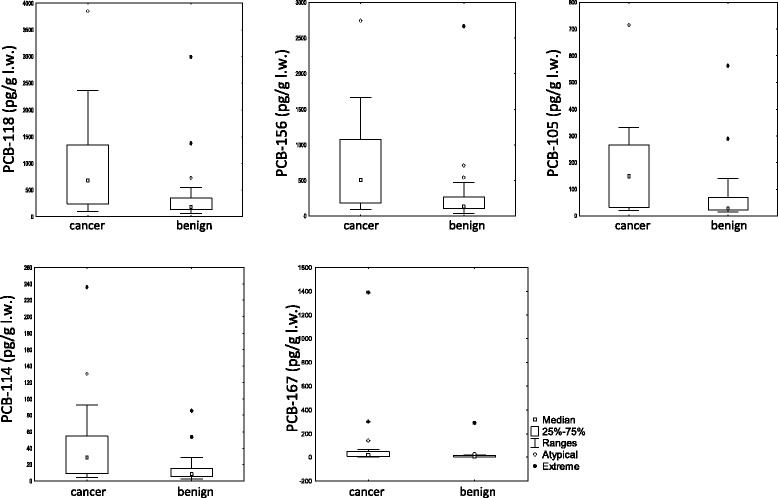
**Distribution of the measurement points represented by “Box and whisker” plots with a confidence interval of 95% in cancer versus benign tumor cases for PCB-118, PCB-156, PCB-105, PCB-114 and PCB-167.**

Subgroups of pet dogs with similar number of individuals and balanced epidemiological criteria were selected. Hence, 54 breast cancers versus 47 no cancer controls were submitted to non-parametric statistical analyses (Mann-Whitney test). 54 breast cancers versus 30 mammary benign tumors were also compared (Table 1). When pollutants are considered together as a toxic equivalent value or as a total amount of congeners, no significant difference is observed inter-groups. However, when statistical analyses are performed for individual molecules, significant differences inside the dl-PCB family restricted to congeners with a non-coplanar chemical structure (mono-ortho type) are found. Notably, PCB-118 and PCB-156 appear almost twice more concentrated in cancer group (median values) than in control groups and these features seem robust when different populations are compared (Table 3). Significant differences observed for PCB-105, PCB-114 and PCB-167 appear group dependant, understanding that Mann-Whitney test tends to generate false negative results. Excluding PCB-167, this shift towards a higher dl-PCB profile seems not due to atypical individuals amongst cancer group but seems rather to correspond to a continuum, as demonstrated by the repartition of cases or controls as a function of dl-PCB concentrations (Figure 2, Table 4). Lastly we can assume on the one hand that all breast cancer cases are probably not related to this chemical exposure, and on the other hand that some of controls may further develop the disease, which is adequate to explain some overlapping measurement points (Figure 3). When focusing on these congeners regardless the dogs’ pathological status but regarding their physical activity as a hypothetic bias, no significant difference were noticed that could mimetic and explain this profile (Table 5). None of the other 88 molecules among the other five chemical families (dioxins, ndl-PCBs, OCPs, BFRs and PFAS) appeared individually associated to mammary cancer, and multivariate analyses did not reveal any synergistic mixture effect.

**Table 3:** **Significant POPs associated to canine mammary cancer**

					**Comparative values**	
					**(p-value <0.05 is significant)**	
	**Relative contribution**	**TEF** ^**2005**^	**Median quantity (pg/g l.w.)**	**Median quantity (pg/g l.w.)**	**p-value (Mann- Whitney)**	**Fold-change**
			Cancer dogs	Non cancer		
			(*n* =54)	dogs (*n* = 47)		
PCB-118	36%	0.00003	359.72	182.89	0.022	1.97
PCB-156	26%	0.00003	271.73	149.28	0.020	1.82
PCB-105	7%	0.00003	56.55	28.40	0.043	1.99
			Cancer dogs	Benign tumors		
			(*n* =54)	(*n* =30)		
PCB-118	36%	0.00003	359.72	182.15	0.020	1.97
PCB-156	26%	0.00003	271.73	133.56	0.017	2.03
PCB-114	1.5%	0.0005	15.43	8.33	0.044	1.85
PCB-167	2%	0.00001	9.28	7.66	0.048	1.21

**Table 4:** **Quartiles of concentrations (pg/g l.w.) for significant dl-PCB congeners established from all dogs (n = 115)**

**pg/g l.w.**	**Q1**	**Q2**	**Q3**	**Q4**
	**Minimum - 25%**	**25% - Median**	**Median - 75%**	**75% - Maximum**
PCB-118	40.3-135.3	135.3-254.4	254.4-540.8	540.8-3910.0
PCB-156	40.7-110.3	110.3-196.6	196.6-404.9	404.9-2742.8
PCB-105	6.0-22.8	22.8-45.7	45.7-90.8	90.8-739.6
PCB-114	1.7-5.9	5.9-12.9	12.9-23.8	23.8-235.7
PCB-167	1.6-5.1	5.1-8.9	8.9-17.1	17.1-1389.4

**Table 5:** **Absence of bias for outdoor physical activity in dl-PCB profiles associated to mammary cancer (p-value >0.05 is non-significant)**

	**Median (pg/g l.w.)**			
	**All dogs**	**Regular or intensive activity ≥ 2h/ week**	**No or scarce activity ≤ 1h/ week**	**p-value** ^**c**^
	**(n = 115)**	**(n = 39)** ^**a**^	**(n = 39)** ^**b**^	**(Mann- Whitney)**
PCB-118	254.4	309.2	252.0	0.430
PCB-156	196.6	212.5	196.6	0.343
PCB-105	45.7	47.0	44.1	0.310
PCB-114	12.9	12.7	10.9	0.284
PCB-167	8.9	9.4	8.5	0.211

^a^Dogs with regular or intense activity.

^b^Dogs with no or scarce activity.

^c^Dogs with regular or intense activity *vs.* Dogs with no or scarce activity.

## 3. Discussion

This innovating project was dedicated to the evaluation of mammary cancer pet dogs in the perspective of an epidemiological model to investigate a potential link between environmental chemical exposure and breast cancer incidence. In our case/control study, we point out some dl-PCB congeners characterized by a mono-ortho structure, namely PCB-118, PCB-156, PCB-105 and PCB-114, as possible candidate markers associated to this pathology. These compounds have already been pointed out in several epidemiological studies focusing on human breast cancer. According to these studies: premenopausal women associated to a higher level of PCB-118, PCB-156 and PCB-105 are between two and three times more likely to develop breast cancer than to be healthy without mammary lesion and without any history of cancer (Demers et al. 2002), whereas such a trend was not noticed in menopause women (Demers et al. 2002; Raaschou-Nielsen et al. 2005; Rusiecki et al. 2004); women associated to a higher level of PCB-118 or PCB-105 are about three times more likely to develop breast cancer than benign breast tumor (Aronson et al. 2000); women associated to a higher level of PCB-156 are between two and four times more likely to develop ER-negative breast cancer subtype than benign breast tumor, whereas such a trend was not noticed in ER-positive subtype (Woolcott et al. 2001); women associated to a higher level of PCB-118 are between three and four times more likely to develop breast cancer recurrence than to be cured 5 years after resection (Muscat et al. 2003). In these studies like in ours, no significant result was observed considering the total concentration of POPs and the TEQ values. Significant features were generally found from specific congeners in particular subgroups of women especially linked to hormonal status or disease outcome, and the statistical power of such restricted population is often a limit to establish clear evidences on etiology or pathogenesis. Numerous polymorphisms on cytochrome P450 (CYP) genes encoding for enzymes playing a key role in the metabolic breakdown of environmental chemicals do exist, which can explain distinctive qualitative profiles of dl-PCB congeners in individuals, despite these molecules were originated from standard industrial mixtures (Aroclors) at baseline (Lind et al. 2014). Some of these P450 polymorphisms have been related to breast cancer (Masson et al. 2005). Furthermore, cumulative POP calculations (based on linear dose-response relationships in toxicological models) are relevant for assess overall levels of exposure and theoretical danger, but not for evaluate the balanced biological responses resulting from competitive binding of numerous exogeneous and endogenous ligands on the AhR in a regulated physiological system (Nebert and Karp 2008; Hao and Whitelaw 2013), as one pollutant can oppose or orientate the effects of the others (Gregoraszczuk et al. 2008; Ohura et al. 2010).

From a mechanistic point of view in the biology of cancer and especially breast cancer, dl-PCB congeners target mainly the AhR, which has been demonstrated to cross-talk with ER signaling (Ohtake et al. 2011) and both might down-regulate breast tumor proliferation by this way and promote tumor escape by ER-negative phenotype expression (Feng et al. 2013). Independently, the AhR interacts also with various oncogenic pathways including those related to the tumor microenvironment such as alteration of extracellular matrix remodeling, promotion of angiogenesis and modulation of inflammation (Feng et al. 2013). These fundamental findings are in accordance with our results suggesting a role for dl-PCB congeners in promoting oncogenesis rather than in initiating mammary lesions, when comparing malignant and benign tumors. Those findings are also in total accordance with clinical data obtained in breast cancer patients: indeed RT-PCR analyses provide evidence that AhR genes are frequently deregulated (Dialyna et al. 2001); its repressor molecule AhRR is also frequently down-regulated by DNA hypermethylation gene silencing (Zudaire et al. 2008); immunohistochemical staining reveals that high levels of AhR is detected in mammary epithelial cells for 87% of the premenopausal breast cancer patients and may contribute to the ER-negative phenotype, whereas the same pattern is not observed in benign tumors (Bidgoli et al. 2010).

All POPs in canine samples were measured at levels largely lower than those reported for human biomonitoring and despite this, the same candidate markers were associated to breast cancer with approximately the same fold-change inter-groups. A focus on dl-PCB congeners - namely PCB-118 and PCB-156 - indicates that their respective concentrations are between 70-150 and 40-80 times less in French dogs (aged 10) in our study than in US or Canadian women (aged 60) at the time of breast cancer diagnosis in studies cited above. However the relative contribution of PCB-118 and PCB-156 inside the dl-PCB family seems more or less conserved between these two species when the whole list of congeners is quantified (Fernandez et al. 2008). This fact supports a major influence of polymorphisms and power balance through competitive binding rather than a toxic critical threshold, unless if the disruption occurred in the womb owing to a similar level of exposure in utero that has changed with age, or unless if the sensitivity of dogs to these molecules is stronger than in humans. Indeed, relative potencies of dioxins and PCBs (TEF values) established from rodents and used by extension for human biomonitoring may turn to be in fact species and congener specific (Sutter et al. 2010). P450 polymorphisms may also exist inter-species. For instance, an intriguing phenomenon is the incapacity of dogs to store any isomers or metabolites of DDT pesticide in all its known forms (o,p’-DDE, p,p’-DDE, o,p’-DDD, p,p’-DDD, o,p’-DDT, p,p’-DDT), whereas p,p’-DDE appears as the main OCP contribution in humans without clearly interfering with breast cancer (Ingber et al. 2013). This canine metabolic specificity that we confirm was established in previous studies performed in Japan, Italy and Pakistan (Kunisue et al. 2005; Storelli et al. 2009; Ali et al. 2013), despite proven petfood contamination. Finally, PCBs hydroxylation ratio (OH-PCBs/PCBs), which is rarely targeted in epidemiological studies, varies also widely in human individuals and in diverse species (Fernandez et al. 2008; Kunisue and Tanabe 2009). This way of interpreting environmental links to breast cancer with the dog model may lead to suggest that decreasing exposure thanks to environmental protective actions may not be effective straight away for human beings, but tends also to suggest that if any, biological effect on breast cancer pathology might be reversible by targeting the AhR (Powell et al. 2013; Safe et al. 2013).

## 4. Conclusions

This study is the first one that evaluates cancer-bearing companion dogs in the perspective of an epidemiological model, in order to investigate a potential link between environmental chemical exposure and breast cancer incidence. We identified pollutants in the dl-PCB family that were already pointed out in several human epidemiological studies on breast cancer (i.e. PCB-118, -156, -105, -114). No significant feature was observed in the other targeted chemical families (dioxins, ndl-PCB, OCPs, BFRs and PFAS). Further biological investigations are required ex vivo to determine if this discrete but recurrent signature could be associated with the etiology and pathogenesis of breast cancer subtypes, or appears as a marker without a role. Such problematic is the same in canine or in human breast cancer.

## Abbreviations

POPs: Persistent organic pollutants
ER: Estrogen receptor
PR: Progesterone receptor
AR: Androgen receptor
OCs: Organochlorine pesticides
ndl-PCBs: Non-dioxin like polychlorobisphenyls
BFRs: Brominated flame retardants
PFAS: Perfluorinated alkylated substances
PCDD/Fs: Dioxins/ furanes
dl-PCBs: Dioxin-like polychlorobisphenyls
AhR: Aryl hydrocarbon receptor
TEQ: Toxic equivalent
TEF: Toxic equivalency factor
